# QSAR Study and Molecular Design of Open-Chain Enaminones as Anticonvulsant Agents

**DOI:** 10.3390/ijms12129354

**Published:** 2011-12-14

**Authors:** Juan C. Garro Martinez, Pablo R. Duchowicz, Mario R. Estrada, Graciela N. Zamarbide, Eduardo A. Castro

**Affiliations:** 1 Department of Chemistry, National University of San Luis, Chacabuco 917, San Luis 5700, Argentine; E-Mails: estrada@unsl.edu.ar (M.R.E.); gzama@unsl.edu.ar (G.N.Z.); 2 INIFTA, (CCT-La Plata-CONICET), Diag. 113 y 64, C.C. 16, Suc.4, La Plata 1900, Argentine; E-Mails: pabloducho@gmail.com (P.R.D.); eacast@gmail.com (E.A.C.)

**Keywords:** QSAR theory, anticonvulsant activity, open-chain enaminone, flexible descriptors

## Abstract

Present work employs the QSAR formalism to predict the *ED*_50_ anticonvulsant activity of ringed-enaminones, in order to apply these relationships for the prediction of unknown open-chain compounds containing the same types of functional groups in their molecular structure. Two different modeling approaches are applied with the purpose of comparing the consistency of our results: (a) the search of molecular descriptors via multivariable linear regressions; and (b) the calculation of flexible descriptors with the CORAL (CORrelation And Logic) program. Among the results found, we propose some potent candidate open-chain enaminones having *ED*_50_ values lower than 10 mg·kg^−1^ for corresponding pharmacological studies. These compounds are classified as Class 1 and Class 2 according to the Anticonvulsant Selection Project.

## 1. Introduction

Enaminones are a group of organic compounds carrying the conjugated system N–C=C–C=O [1]. The literature reports information about the chemistry of enaminones, their physicochemical properties and biological activities [2–10]. In spite of the interest in these compounds, only a limited number of theoretical works have been published on the prototype enaminone 2-propenal-3-amine based on the semiempirical molecular orbitals theory [11,12] and the quantum chemical study using *ab initio* method or the density functional theory [13–15].

**Figure 1:**
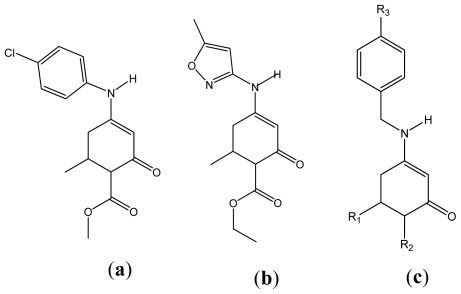
(**a**) Aniline enaminone derivative DM5. (**b**) Isoxasol enaminone derivative ON2. (**c**) Benzylamine enaminone derivative.

Biologically active enaminones may be classified in two different types, according to the layout of the functional group [13–15]: (a) open-chain enaminones (OCEs), where the characteristic group is part of a chain (thus having the flexibility that enables different conformers); and (b) ringed enaminones (REs), where the characteristic group is part of a ring and the enaminone group is not flexible. In recent years, a group of REs has been reported as anticonvulsant. The mechanism of action of these biomolecules would be similar to many classic antiepileptics and second-generation drugs, while they act on ion channels by blocking the passage of ions through them [2–10]. Among the bioactive REs appears DM5 (methyl 4-(4-chlorophenylamino), 6-methyl,2-oxocyclohex-3-ene carboxylate), (Figure 1a) and ON2 (ethyl 6-methyl,4-(5-methylisoxazol-3-ylamino), 2-oxocyclohex-3-ene carboxylate), (Figure 1b) [6,7]. Another family of enaminones with biological activity is derived from benzylamine enaminones, (Figure 1c) [9]. These have anticonvulsant activity similar to DM5 (aniline enaminone derivate) and ON2 (isoxasol enaminone derivate).

Distance between the carbonyl oxygen and the aromatic ring is of great importance during the binding of the molecule with the sodium channel [16]. Conformations that adopt a RE influence this distance may result in different activities [2–9]. In a previous study, we have performed a QSAR study on the activity of various RE in the active conformation [17].

**Figure 2:**
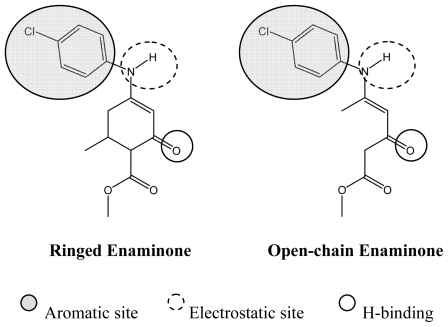
Pharmacophore models reported in the literature and ringed and open-chain enaminones structures.

Now, a comparison between both enaminone families demonstrates the similarity of the molecular structure and functional groups involved in the linkage with the sodium channel, as evidenced by the different pharmacophore models reported in the literature [16,18–20] (Figure 2). In this way, an OCE could bind to the receptor in a similar way as the REs do. Moreover for the OCE, the flexible open chain and greater ability to transport through biological membranes would allow more precise fitting of its site of action.

Accordingly, it is feasible to formulate the following question: could an open-chain enaminone have anticonvulsant activity as it is the case for ringed enaminones? Several techniques have been developed to elucidate a relationship between the structure and biological activity, SAR, QSAR [21], S-SAR [22–24]. The main objective of this work is to study a molecular set of OCEs for predicting their antiepileptic activity using the QSAR methodology, which would allow us to provide some guidelines on the anticonvulsant properties of this class of molecules.

## 2. Materials and Methods

### 2.1. Experimental Data

The experimental information on the antiepileptic activities of the molecular structures is obtained from various recent publications, by methods that have been previously reported [4–10]. Due to the scarcity of experimental information and the need for QSAR models, it is necessary to collect data from different authors [4–10]. However, we pay attention that the parameter of activity (*ED*_50_), which represents the dose at which 50% of individuals reach the desired effect, is obtained by using the same assay. This is determined in the “Anticonvulsant Selection Project” (ASP) by the experimental method “Maximal electroshock seizure” (MES) [2,7,8,25]. For modeling purposes, we use Log_10_ *ED*_50_ to get a more standardized property.

### 2.2. Geometry Optimization and Molecular Descriptors Calculation

The structures of all the examined compounds are optimized with the Semiempirical Method PM3 (Parametric Method-3) included in the HyperChem 6.03 software [26]. By means of the software Dragon [27], we calculate a set of 1307 molecular descriptors [28], which includes. 0D: Constitutional Descriptors, 1D: Functional Groups, Empirics Descriptors, Atom Centred Fragments; 2D: Descriptors topological, Molecular walk counts, Galvez Charge Index, BCUT Descriptors; 3D: Descriptors of Charge, aromatic index, molecular profiles of Randic, Geometry Descriptors, RDF Descriptors, 3D-Morse Descriptors, WHIM descriptors and GATEWAY Descriptors. In addition, 5 descriptors obtained from the semiempirical calculation are added (molecular dipole moment, energy of the HOMO and LUMO and HOMO-LUMO gap). Therefore, the set of descriptors contains *D* = 1312 variables.

### 2.3. Model Development

The QSAR established in this work are obtained via two different modeling approaches with the purpose of comparing the consistency of our results: (a) the search of molecular descriptors via multivariable linear regressions; and (b) the calculation of flexible descriptors with the CORAL (CORrelation And Logic) program.

#### 2.3.1. Linear Descriptors Search

In the search for the best model we use the Matlab 7.0 [29]. Our quest is to find from the set of *D* descriptors a subset of *d* ones (*d* <<< *D*) with the minimum standard deviation (*S*), so we use the Replacement Method (RM) [30–32]. Standard deviation is defined as follows:

(1) $$S=\frac{1}{\left(N-d-1\right)}\sum_{i=1}^{N}{{res}_{i}}^{2}$$

where *N* is the number of molecules in the calibration set CC (molecular set used for calibration of the model), *res_i_* is the residue of the molecule *i* (difference between experimental and predicted property of *i*).

The QSAR Theory searches for the best predictions of the activity, but it is a rule in practice that the models should be simple, interpretable, and have a descriptor per six or seven molecules in order to achieve satisfactory results [33]. Then, we calculate the maximum number of descriptors (*d*_nm_) to be included in the linear regression equation as:

(2) $$d_{\text{nm}}=\frac{N}{7}$$

On the other hand, the Kubinyi function *FIT* [34,35] is used to get the optimum number of descriptors (*d*_opt_) of each linear regression established. The *FIT* criterion is a very effective method for obtaining the optimal number of descriptors of a particular model [32–34].

#### 2.3.2. Calculation of Flexible Descriptors

CHEMPREDICT/CORAL (CORrelation And Logic) version 1.4 [36] is a freeware for Windows. Each molecular structure must be represented by SMILES (Simplified Molecular Input Line Entry System) notation, calculated with ACD/ChemSketch software [37]. CORAL approach is based on the presence of certain SMILES attributes occurring in the molecule which can be associated to the activity of the molecule under evaluation [38–41]. As SMILES attributes are used the symbols representing the chemical elements, cycles, branching of molecular skeleton, charges, *etc.* More specific details on the CORAL algorithm can be found in the recent literature [38–41].

#### 2.3.3. Model Validation

A next step of current analysis is to verify the validation (predictive capability) of the QSAR relationships established on a calibration set of chemical structures. These must be predictive and capable to adapt equally-well on new structures (test set) that do not participate during the training of the model. We choose the well-known leave-one-out (loo) and leave-more-out (l-%-o) cross-validation procedures, where % represents the percentage of molecules removed from the calibration set. For l-%-o, we generate 1,000,000 cases of random molecules removal, where % = 10 (five compounds). The standard deviations *S*_test_ and *S*_l-%-o_ are calculated in this step.

## 3. Results and Discussion

### 3.1. QSAR on Ringed-Enaminones

In a previous work we have developed a mathematical model for the prediction of *ED*_50_ in REs compounds [17]. This model contains five molecular descriptors and involves a calibration set of 46 compounds. For such model (Equation 3), validation is performed with a set of five molecules, leading to *S*_test_ = 0.232 and *R*_test_ = 0.835:

(3) $$\begin{array}{l} {\text{log}}_{10}\;{ED}_{50}=-3.3102(\pm 0.579)+3.7124(\pm 0,737)BELe6-2.3384(\pm 0.387)BELp8+\\ 0.1282(\pm 0.017)RDF025v+0.66732(\pm 0.118)Mor15e+33.683.(\pm 5.16)R4e+ \end{array}$$

$$\begin{array}{c} N=46;p<10^{-4};R_{\text{cal}}=0.870;S_{\text{cal}}=0.206;R_{\text{test}}=0.835;S_{\text{test}}=0.232;R_{\text{loo}}=0,925;\\ S_{\text{loo}}=0.198;R_{1-10-\text{o}}=0.712;S_{1-10-\text{o}}=0.319 \end{array}$$

In this study, we propose a new five-descriptor model (Equation 4). The calibration is established with 51 compounds, including all compounds belonging to Equation 3. Thus, Equation 4 contains more biochemical information and its predictive power may be higher. This last model is applied to the same calibration and test sets of Equation 3, leading to:

(4) $$\begin{array}{l} {\text{log}}_{10}\;{ED}_{50}=2.247(\pm 0.867)-0.024(\pm 0.005)G(O\ldots Cl)+0.0072(\pm 0.014)RDF025m-\\ 0.238(\pm 0.044)\;RDF115m+35.631(\pm 4.793)R4e^{+}+0.402(\pm 0.076)\Delta E_{Homo-Lumo} \end{array}$$

$$\begin{array}{c} N=51;p<10^{-4};R_{\text{cal}}=0.864;S_{\text{cal}}=0.209;R_{\text{test}}=0.947;S_{\text{test}}=0.204;R_{\text{loo}}=0.847;\\ S_{\text{loo}}=0.228;R_{1-\%-\text{o}}=0.746;S_{1-\%-\text{o}}=0.343 \end{array}$$

In Equation 3, *BELe6* and *BELp8* are BCUT descriptors, *RDF025v* is a Radial Distribution Function descriptor, *Mor15e* is a 3D-MoRSE descriptor and *R4e^+^* is a 3D GATEWAY descriptor. The structural variables appearing in Equation 4 combine multidimensional aspects of the molecular structure and are classified as follows: Radial Distribution Function descriptors (*RDF025m* and *RDF115m*), Geometrical (*G(O..Cl)*), GATEWAY (*R4e^+^*) and HOMO-LUMO energy gap (*Homo-Lumo*). A brief explanation of the descriptors participating in both equations is provided in Table 1.

The highest intercorrelation coefficient for the five descriptors of Equation 3 is 0.733. This is because *BELe6* and *BELp8* descriptors belong to the same BCUT family. In general, QSAR models accept intercorrelations up to the value 0.98, but the orthogonalization process can be used to give better analysis when necessary [42,43]. Equation 4 has low intercorrelations between descriptors, the highest value is 0.561. Only descriptor *R4e^+^* (*R* maximal autocorrelation of lag 4/weighted by atomic Sanderson Electronegativities) simultaneously appears in both equations and has low intercorrelations to the remaining ones.

**Figure 3:**
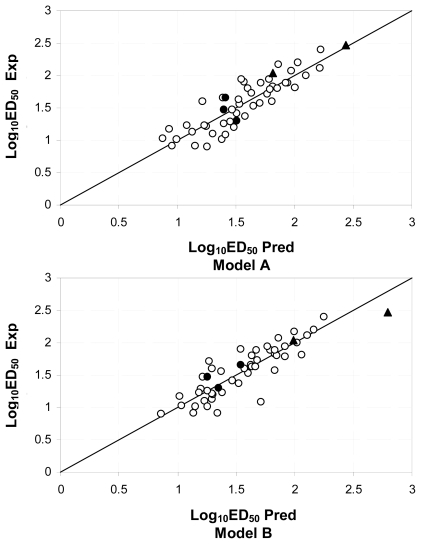
Experimental and predicted Log_10_ *ED*_50_ plot. ○ Calibration set ● test set ▴ Enaminones of test set.

Table 2 lists the compounds of both models, together with the experimental and predicted *ED*_50_ values. Figure 3 shows the experimental and predicted Log_10_ *ED*_50_ plot for the calibration and validation sets. From this figure it can be noted that the two enaminones of the validation set, **47** and **51**, are very well predicted. Dispersion plots of the residuals for the calibration and test sets are provided in the supplementary material. Such figures reveal that the behavior of the residuals in terms of the predictions follows a random distribution, in accordance to the assumption involved in linear regression analysis. No molecule in the set exhibits a residual larger than the value of S.

Now, it is feasible to improve the statistical performance of Equations 3 and 4 by using models established via flexible descriptor definitions calculated with the CORAL program. We run a Monte Carlo simulation for obtaining the *DCW*^3^ descriptor of Equation 5, achieving the following QSAR model:

(5) $$\begin{array}{c} {Log}_{10}{ED}_{50}=-0.1906(\pm 0.0227)+0.069(\pm 0.0008){DCW}^{3}\\ N=46;p<10^{-4};R_{\text{cal}}=0.7627;S_{\text{cal}}=0.192;R_{\text{loo}}=0.6998;S_{\text{loo}}=0.350 \end{array}$$

The specification of the numerical parameters used in the CORAL calculation is: number of epochs: 40, number of probes: 5, range of threshold values: 0–2, *D*_start_ = 0.1, *d*_precision_ = 0.001, d*R*_weight_ = 0, d*C*_weight_ = 0, threshold range = 0–5, and *α* = *β* = 0.

**Figure 4:**
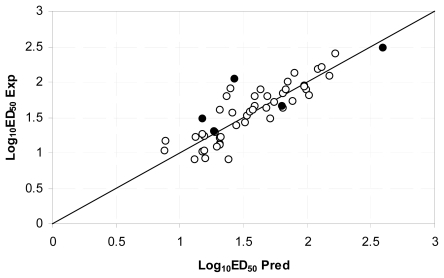
Experimental and predicted Log_10_ *ED*_50_ plot using flexible descriptors model: ○ Calibration set ● test set.

Figure 4 plots the predicted activities as function of the experimental data. The predictions achieved by model 5 are included in Table 2.

It is easily appreciated from the statistical parameters of calibration and leave-one-out validation that the quality of Equations 3 and 4 outperforms that of Equation 5. However, we decide to include Equation 5 in order to compare the predictions.

Another crucial problem to consider is the definition of the Applicability Domain (AD) of a QSAR model [44–46]. In other words, not even a robust, significant, and validated QSAR model can be expected to reliably predict the modeled property for the entire universe of molecules. In fact, only the predictions for molecules falling within this AD can be considered reliable and not just model extrapolations. The AD is a theoretical region in chemical space, and depends upon the set of chemical structures and the experimental property analyzed; hence the AD is different for each QSAR model established. We define the AD for each QSAR in terms of the ranges of variation of the numerical values of its descriptors: a molecular structure would be, in principle, reliably predicted if its numerical descriptor values fall within such ranges. Thus, for Equation (3) *BELe6*: [0.7180–1.0260], *BELp8*: [0.4540–1.0870], *RDF025v*: [12.2150–22.5420], *Mor15e*: [−0.6920–27.8150], *R4e^+^*: [0.0330–0.0710]; for Equation (4) *G(O...Cl)*: [0.0000–32.1200], *RDF025m*: [13.9580–24.1070], *RDF115m*: [0.0000–4.8410], *R4e^+^*: [0.0330–0.0710], *ΔE_Homo-Lumo_*: [−9.8192–(−7.8810)]; for Equation (5) *DCW*^3^: [15.5091–40.4327]. In addition, the predicted activity for a considered structure based on a given combination of descriptors should fall inside (or close to) the range of the experimental activity variation, which in the present case is Log_10_ *ED*_50_: [0.8970–2.4770].

### 3.2. QSAR on Open-Chain Enaminones

**Figure 5:**
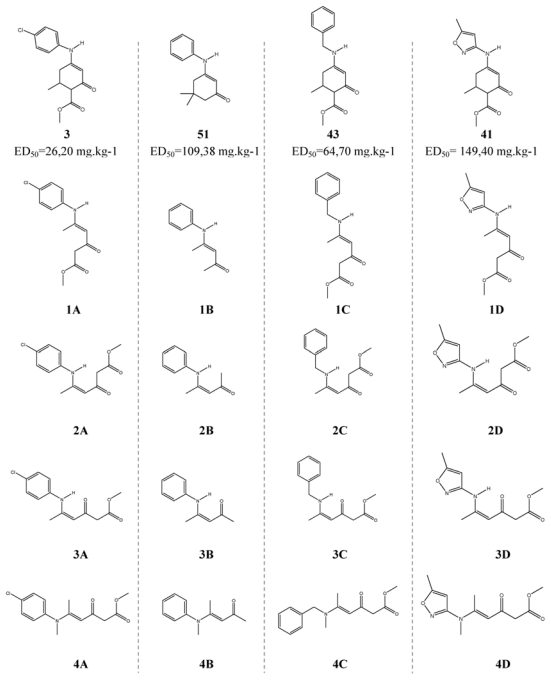
Structure of the 16 conformers of open-chain enaminones. Scheme for the selection of the compounds.

The selected OCEs are structurally-related to the REs used in the calibration and validation sets. For this selection, an analysis of molecular modulation is carried out, based on an active molecule. Then, the molecules **1A**, **1B**, **1C** and **1D** are obtained from molecules **3**, **51**, **43** and **41** (Figure 5). This figure shows the conformers of the OCEs. Molecules **3** and **51** belong to the family of aniline derivatives, **43** pertains to the family of benzylamine derivatives and **41** belongs to the family of isoxasol derivatives.

The structural similarity between the molecules used in the models and the OCEs suggests that the models developed in this work would serve to predict *ED*_50_ of these molecules. Having no experimental values, a way to verify the predictions is to note that Equations 3 and 4 do not lead to absurd predictions (different predictions for the same molecules). As shown in Table 3, the predictions are similar for both models. Both equations predict that **1B** is the most active, while the enaminone with lower activity is **3A**. Then, we argue that the predictions obtained are not at random, and that the predicted values of *ED*_50_ obtained with both models should be close to the experimental observations.

## 4. Conclusions

A linear QSAR model is developed to predict *ED*_50_ in REs and applied for the prediction of OCEs. In addition, an alternative linear model using a different methodology based on the flexible descriptor definition is obtained with the same purpose. The developed models allow the prediction of antiepileptic activities of 16 OCEs. These compounds are presented as candidate structures for corresponding pharmacological studies. The 16 enaminones would be classified as Class 1 and Class 2 according to ASP. Several of the *ED*_50_ obtained here are less than 10 mg·kg^−1^. Accordingly, conformational flexibility in OCEs is a crucial factor to be considered during the study of the antiepileptic activity behaviour.

## Supplementary Material



## Figures and Tables

**Table 1:** Symbols and description for molecular descriptors involved in QSAR.

Descriptor	Type	Details
*BELe6*	BCUT	Lowest eigenvalue *n*. 6 of Burden matrix/weighted by atomic Sanderson electronegativities
*BELp8*		Lowest eigenvalue *n*. 8 of Burden matrix/weighted by atomic polarizabilities
*RDF025v*	Radial Distribution Function	Radial Distribution Function—2.5/weighted by atomic van der Waals volumes
*RDF025m*		Radial Distribution Function—2.5/weighted by atomic masses
*RDF115m*		Radial Distribution Function—11.5/weighted by atomic masses
*Mor15e*	3D-MoRSE	3D-MoRSE—signal 15/weighted by atomic Sanderson electronegativities
*R4e^+^*	GETAWAY	*R* maximal autocorrelation of lag 4/weighted by atomic Sanderson electronegativities
*G(O..Cl)*	Geometrical	Sum of geometrical distances between O..Cl
*Homo-Lumo*	Quantum Chemical	HOMO-LUMO energy gap

**Table 2:** Experimental and predicted Log_10_ *ED*_50_ antiepileptic activity values of the compounds of calibration set and test set.

No.	Chemical name	*ED*_50_ (mg·Kg^−1^)	Exp.	Equation 3	Equation 4	Equation 5
**1**	Ethyl 6-methyl-4-(5-methylisoxazol-3-ylamino)-2-oxocyclohex-3-enecarboxylate	68.39 [4]	1.835	1.815	1.831	1.813
**2**	Methyl 4-(4-cyanophenylamino)-6-methyl-2-oxocyclohex-3-enecarboxylate	248.31 [4]	2.395	2.229	2.252	2.226
**3**	Methyl 4-(4-chlorophenylamino)-6-methyl-2-oxocyclohex-3-enecarboxylate	26.18 [4]	1.418	1.509	1.466	1.517
**4**	2-acetamido-*N*-benzylpropanamide	76.38 [5]	1.883	1.716	1.677	1.634
**5**	2-acetamido-*N*-(3-fluorobenzyl)propanamide	77.27 [5]	1.888	1.945	1.791	1.994
**6**	2-acetamido-*N*-(2-fluorobenzyl)-2-(furan-2-yl)acetamide	39.99 [5]	1.602	1.215	1.294	1.315
**7**	2-acetamido-*N*-(3-fluorobenzyl)-2-(furan-2-yl)acetamide	13.27 [5]	1.123	1.132	1.291	1.315
**8**	2-acetamido-*N*-(4-fluorobenzyl)-2-(furan-2-yl)acetamide	12.68 [5]	1.103	1.302	1.228	1.315
**9**	2-acetamido-*N*-(2,5-difluorobenzyl)-2-(furan-2-yl)acetamida	23.77 [5]	1.376	1.577	1.522	1.448
**10**	2-acetamido-*N*-(2,6-difluorobenzyl)-2-(furan-2-yl)acetamide	62.95 [5]	1.799	1.604	1.631	1.687
**11**	2-acetamido-*N*-benzylpent-4-enamide	33.57 [5]	1.526	1.653	1.605	1.533
**12**	2-acetamido-*N*-benzyl-2-(tetrahydrofuran-2-yl)acetamide	51.64 [5]	1.713	1.770	1.272	1.746
**13**	2-acetamido-*N*-benzyl-2-(furan-2-yl)acetamide	10.28 [5]	1.012	1.383	1.252	1.182
**14**	2-acetamido-*N*-benzyl-2-(5-methylfuran-2-yl)acetamide	19.19 [5]	1.283	1.450	1.200	1.282
**15**	2-acetamido-*N*-benzyl-2-(1H-pyrrol-2-yl)acetamide	16.07 [5]	1.206	1.486	1.299	1.315
**16**	2-acetamido-*N*-benzyl-2-(5-methyl-1H-pyrrol-2-yl)acetamide	36.48 [5]	1.562	1.530	1.376	1.415
**17**	2-acetamido-*N*-benzyl-2-(thiophen-2-yl)acetamide	44.77 [5]	1.651	1.388	1.628	1.593
**18**	2-acetamido-*N*-benzyl-2-(thiophen-3-yl)acetamida	87.70 [5]	1.943	1.783	1.770	1.979
**19**	2-acetamido-*N*-benzyl-2-(1H-pyrrol-1-yl)acetamide	80.17 [5]	1.904	1.572	1.538	1.399
**20**	2-acetamido-*N*-benzyl-2-(1H-pyrazol-1-yl)acetamide	16.48 [5]	1.217	1.249	1.294	1.325
**21**	2-acetamido-*N*-benzyl-2-(pyridin-2-yl)acetamide	10.79 [5]	1.033	0.880	1.037	1.195
**22**	2-acetamido-3-amino-*N*-benzyl-3-thioxopropanamide	86.50 [5]	1.937	1.550	1.921	1.981
**23**	2-acetamido-*N*-benzyl-2-(ethylamino)acetamide	42.36 [5]	1.627	1.525	1.635	1.679
**24**	2-acetamido-*N*-benzyl-2-(hydroxy(methyl)amino)acetamide	29.99 [5]	1.477	1.465	1.215	1.712
**25**	2-acetamido-*N*-benzyl-2-(1-phenylhydrazinyl)acetamide	42.76 [5]	1.631	1.524	1.663	1.811
**26**	2-acetamido-*N*-benzyl-2-ethoxyacetamide	61.94 [5]	1.792	1.795	1.922	1.368
**27**	2-acetamido-*N*-benzyl-3-methoxypropanamide	8.30 [5]	0.919	0.954	1.135	1.201
**28**	2-acetamido-*N*-benzyl-3-ethoxypropanamide	16.98 [5]	1.230	1.232	1.385	1.197
**29**	2-acetamido-*N*-benzyl-2-(pyrazin-2-yl)acetamide	14.79 [5]	1.170	0.929	1.015	0.893
**30**	2-acetamido-*N*-benzyl-2-(pyrimidin-2-yl)acetamida	8.09 [5]	0.908	1.151	1.344	1.121
**31**	2-acetamido-*N*-benzyl-2-(oxazol-5-yl)acetamide	10.50 [5]	1.021	0.998	1.149	0.88
**32**	2-acetamido-*N*-benzyl-2-(thiazol-5-yl)acetamide	11.99 [5]	1.079	1.417	1.717	1.291
**33**	2-acetamido-2-(3-aminophenylamino)-*N*-benzylacetamide	98.40 [5]	1.993	2.102	2.023	1.85
**34**	2-acetamido-*N*-benzyl-2-(furan-2-yl)acetamide	18.37 [5]	1.264	1.396	1.255	1.182
**35**	Ethyl 4-(4-chlorophenylamino)-6-methyl-2-oxo-3-cyclohexene-1-carboxylate	16.67 [7]	1.222	1.085	1.184	1.124
**36**	Ethyl 4-(4-bromophenylamino)-6-methyl-2-oxo-3-cyclohexene-1-carboxylate	7.89 [7]	0.897	1.259	0.861	1.383
**37**	Ethyl 6-methyl-2-oxo-4-(4-(trifluoromethoxy)phenylamino)cyclohex-3-enecarboxylate	37.07 [7]	1.569	1.708	1.831	1.553
**38**	Ethyl 4-(4-cianophenylamino)-6-methyl-2-oxo-3-cyclohexene-1-carboxylate	63.10 [7]	1.800	1.852	1.847	1.595
**39**	3-(4-chlorophenylamino)-5-methyl-2-cyclohexenone	40.36 [7]	1.606	1.804	1.570	1.576
**40**	3-(4-iodophenylamino)-5-methyl-2-cyclohexenone	76.91 [7]	1.886	1.924	1.829	1.835
**41**	Methyl 6-methyl-4-(5-methylisoxazol-3-ylamino)-2-oxocyclohex-3-cyclohexene-1-carboxylate	149.28 [8]	2.174	1.867	2.001	2.087
**42**	*Tert*-butyl 6-methyl-4-(5-methylisoxazol-3-ylamino)-2-oxocyclohex-3-cyclohexene-1-carboxylate	119.67 [8]	2.078	1.974	1.861	2.181
**43**	Methyl 4-(benzylamino)-6-methyl-2-oxocyclohex-3-cyclohexene-1-carboxylate	64.57 [9]	1.810	2.005	2.062	2.019
**44**	Methyl 4-(4-fluorobenzylamino)-6-methyl-2-oxocyclohex-3-cyclohexene-1-carboxylate	158.85 [9]	2.201	2.030	2.164	2.118
**45**	3-(benzylamino)-5,5-dimethylcyclohex-2-cyclohexenone	52.97 [9]	1.724	1.633	1.678	1.892
**46**	Methyl 4-(benzylamino)-6,6-dimethyl-2-oxocyclohex-3-enecarboxylate	131.83 [10]	2.120	2.219	2.107	1.904
47*	Methyl 6-methyl-4-(4-nitrophenylamino)-2-oxocyclohex-3-enecarboxylate	299.92 [4]	2.477	2.441	2.793	2.599
48*	2-acetamido-*N*-benzyl-2-phenylacetamide	20.28 [7]	1.307	1.505	1.347	1.269
49*	2-acetamido-*N*-benzyl-2-(dimethylamino)acetamide	45.29 [7]	1.656	1.413	1.540	1.804
50*	2-acetamido-2-(furan-2-yl)-*N*-(pyridin-3-ylmethyl)acetamide	29.99 [7]	1.477	1.396	1.255	1.182
51*	5,5-dimethyl-3-(phenylamino)cyclohex-2-enone	109.14 [10]	2.038	1.812	1.990	1.434

* Molecules of test set.

**Table 3:** Log_10_ *ED*_50_ for open-chain enaminones predicted by Equations 3 and 4.

Molecule	Equation 3	Equation 4	PSA a
**1A**	2.051	2.041	2
**2A**	2.426	2.093	2
**3A**	2.456	2.229	2
**4A**	2.323	2.037	2
**1B**	0.956	0.245	1
**2B**	1.529	0.903	1
**3B**	1.103	0.667	1
**4B**	1.393	0.758	1
**1C**	1.329	1.241	1
**2C**	1.462	1.556	1
**3C**	1.794	1.446	1
**4C**	1.505	1.177	1
**1D**	1.295	1.416	1
**2D**	1.583	1.375	1
**3D**	2.172	1.980	b
**4D**	1.291	1.183	1

a Anticonvulsant Screening Project (ASP) (21). Class 1: anticonvulsant activity at 100 mg·kg^−1^ or less; Class 2: anticonvulsant activity at doses greater than 100 mg·kg^−1^; Class 3: inactive at doses of 300 mg·kg^−1^.

b Equation 4: Class 2; Equation 5: Class 1 (95 mg·kg^−1^).
