# Supplementary material for: QSAR Study and Molecular Design of Open-Chain Enaminones as Anticonvulsant Agents
Source: Int J Mol Sci. 2011 Dec 14;12(12):9354–68. doi: 10.3390/ijms12129354 (PMC3257134; doi:10.3390/ijms12129354)

Table 1S. Correlation matrix of Eq. 3&4.

Eq. 3	***BELe*6**	***BELp*8**	***RDF*025*v***	***Mor*15*e***	***R*4*e*** $^{+}$
***BELe*6**	1	0.733	0.528	0.022	0.355
***BELp*8**		1	0.445	0.150	0.432
RDF*025*v			1	0.514	0.227
Mor*15*e				1	0.284
R*4*e $^{+}$					1
Eq.4	***G(O..Cl)***	***RDF*025*m***	***RDF*115*m***	***R*4*e*** $^{+}$	$\Delta E_{\text{homo-lumo}}$
G(O..Cl)	1	0.129	0.027	0.058	0.295
RDF*025*m		1	0.219	0.255	0.561
RDF*115*m			1	0.040	0.371
R*4*e $^{+}$				1	0.128
$\Delta E_{\text{homo-lumo}}$					1

Table 2S. Values for molecular descriptors involved in the QSAR models.

Molecule	G(O..Cl)	RDF025m	RDF115m	R4e+	ΔE $_{Homo-Lumo}$	BELe6	BELp8	RDF025v	Mor15e	R4e+	DCW
**1**	0	18.148	0.205	0.049	-8.4927	1.003	0.925	15.937	-0.193	0.049	29.041067
**2**	0	22.111	0.764	0.054	-8.2642	0.914	0.944	20.238	-0.089	0.054	35.0239432
**3**	32.12	20.508	0.016	0.054	-8.4372	0.807	0.938	18.597	-0.278	0.054	24.7536715
**4**	0	15.167	0.089	0.063	-9.6534	0.723	0.655	13.392	0.053	0.063	26.4416034
**5**	0	13.958	0.016	0.067	-9.552	0.718	0.51	12.215	-0.06	0.067	31.6547677
**6**	0	16.736	0.003	0.043	-9.1635	0.837	0.893	13.731	0.446	0.043	21.8217023
**7**	0	16.879	0.003	0.044	-9.2842	0.837	0.893	13.863	0.247	0.044	21.8217023
**8**	0	16.67	0.781	0.047	-9.2108	0.829	0.894	13.64	0.441	0.047	21.8217023
**9**	0	17.487	0.119	0.046	-8.928	0.837	0.893	13.823	0.82	0.046	23.7517958
**10**	0	17.425	0.006	0.048	-8.8889	0.835	0.894	13.819	0.775	0.048	27.2141538
**11**	0	16.73	0.007	0.058	-9.7147	0.807	0.8	15.273	-0.11	0.058	24.9866057
**12**	0	17.734	0.006	0.047	-9.7484	0.866	0.896	15.359	0.613	0.047	28.067946
**13**	0	15.721	0	0.047	-9.4436	0.839	0.892	13.338	0.557	0.047	19.8916089
**14**	0	17.214	0.013	0.04	-9.2114	0.903	0.923	14.139	0.609	0.04	21.3492188
**15**	0	16.119	0	0.045	-9.2193	0.889	0.895	13.727	0.47	0.045	21.8164011
**16**	0	17.483	0.141	0.043	-9.0118	0.912	0.976	14.67	0.612	0.043	23.274011
**17**	0	19.502	0.011	0.047	-9.1749	0.858	0.882	16.157	-0.117	0.047	25.8501806
**18**	0	20.749	0.007	0.049	-9.2239	0.888	0.891	17.253	0.027	0.049	31.4398952
**19**	0	16.429	0.009	0.048	-8.9421	0.894	0.894	13.798	0.402	0.048	23.0391821
**20**	0	17.108	0.006	0.049	-9.7608	0.842	0.892	13.682	0.173	0.049	21.9634732
**21**	0	17.87	0.223	0.039	-9.5188	0.807	0.906	16.02	-0.081	0.039	20.079641
**22**	0	16.131	0	0.048	-7.9399	0.803	0.673	14.293	0.006	0.048	31.472777
**23**	0	17.677	0.011	0.056	-9.6315	0.807	0.831	15.397	-0.116	0.056	27.0951263
**24**	0	16.586	0.095	0.048	-9.7205	0.807	0.799	13.35	0.479	0.048	27.5787326
**25**	0	23.441	0	0.036	-8.8231	0.827	0.976	20.033	0.398	0.036	29.0036728

**26**	0	14.609	0.088	0.071	-9.6537	0.807	0.76	13.289	-0.312	0.071	22.5866954
**27**	0	15.202	0	0.049	-9.8192	0.807	0.851	13.415	-0.167	0.049	20.1719096
**28**	0	15.755	0.012	0.053	-9.6423	0.807	0.882	14.216	0.003	0.053	20.1125542
**29**	0	17.962	0.236	0.033	-9.0529	0.807	0.877	15.137	0.364	0.033	15.6971736
**30**	0	18.099	0.126	0.041	-9.0313	0.807	0.848	15.132	0.192	0.041	19.0039321
**31**	0	15.702	0.017	0.047	-9.6855	0.838	0.892	13.334	-0.013	0.047	15.5091415
**32**	0	19.01	0.06	0.048	-8.927	0.855	0.882	15.509	0.016	0.048	21.4677132
**33**	0	23.297	0	0.046	-8.788	0.911	0.976	20.053	0.289	0.046	29.5695789
**34**	0	15.737	0	0.047	-9.438	0.839	0.892	13.371	0.57	0.047	19.8916089
**35**	29.93	20.231	0.757	0.047	-8.1603	0.807	0.942	18.874	-0.6	0.047	19.057103
**36**	0	19.618	4.841	0.047	-8.2445	0.807	0.937	18.624	-0.308	0.047	22.8104277
**37**	0	21.96	0.678	0.041	-8.1818	1.026	0.976	20.073	-0.692	0.041	25.2712115
**38**	0	22.021	1.532	0.045	-8.002	0.994	0.976	20.725	-0.626	0.045	25.8797197
**39**	10.38	16.408	0	0.048	-8.2366	0.746	0.474	15.814	-0.285	0.048	25.609845
**40**	0	15.712	0	0.048	-8.0945	0.758	0.454	15.465	-0.175	0.048	29.3631697
**41**	0	18.648	0.047	0.055	-8.7837	0.906	0.924	15.53	0.197	0.055	33.0021628
**42**	0	24.107	0.238	0.039	-8.5742	1.012	1.087	20.426	0.206	0.039	34.368035
**43**	0	20.767	0.083	0.053	-8.8111	0.907	0.943	20.045	-0.301	0.053	32.0250065
**44**	0	21.769	0.006	0.052	-8.6927	0.903	0.943	20.452	-0.27	0.052	33.4566331
**45**	0	19.883	0	0.044	-8.8601	0.807	0.812	20.362	-0.369	0.044	30.1893965
**46**	0	23.49	0	0.048	-8.7908	0.911	0.999	22.542	-0.034	0.048	30.3611176
**47**	0	22.933	0.729	0.063	-7.881	0.935	0.941	20.184	27.815	0.063	40.4327202
**48**	0	19.385	0.006	0.043	-9.5012	0.807	0.918	18.103	25.9	0.043	21.1553498
**49**	0	17.615	0	0.051	-9.4182	0.807	0.898	15.249	25.331	0.051	28.900144
**50**	0	15.737	0	0.047	-9.438	0.839	0.892	13.371	23.865	0.047	19.8916089
**51**	0	18.771	0	0.05	-8.4155	0.779	0.789	18.688	21.47	0.05	23.5480436

Figure 1S. Dispersion plot of the residuals for the ○ Calibration set and ▲ test set according to Eq. 3.

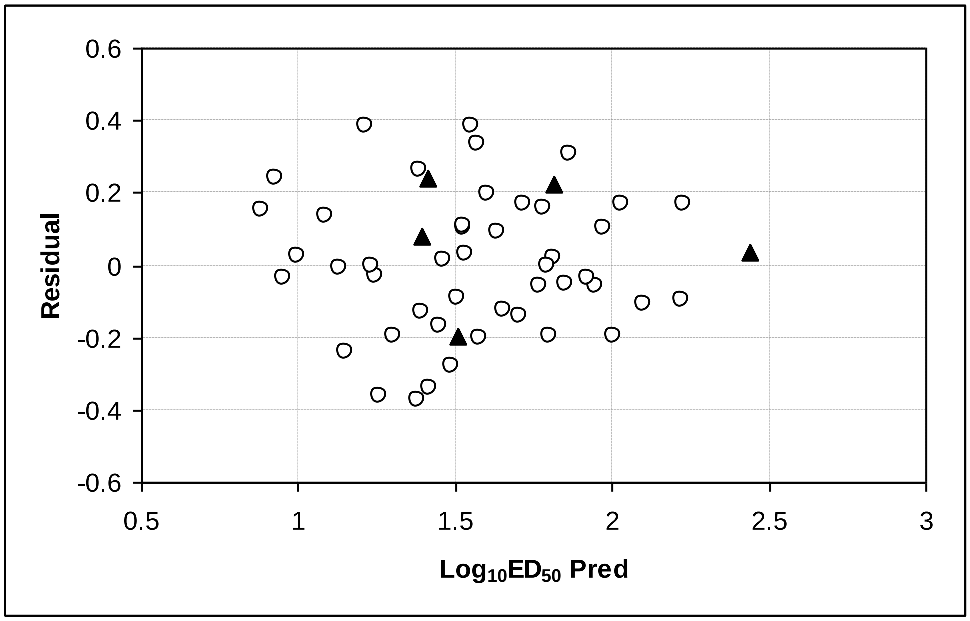

Figure 2S. Dispersion plot of the residuals for the ○ Calibration set and ▲ test set according to Eq. 4.

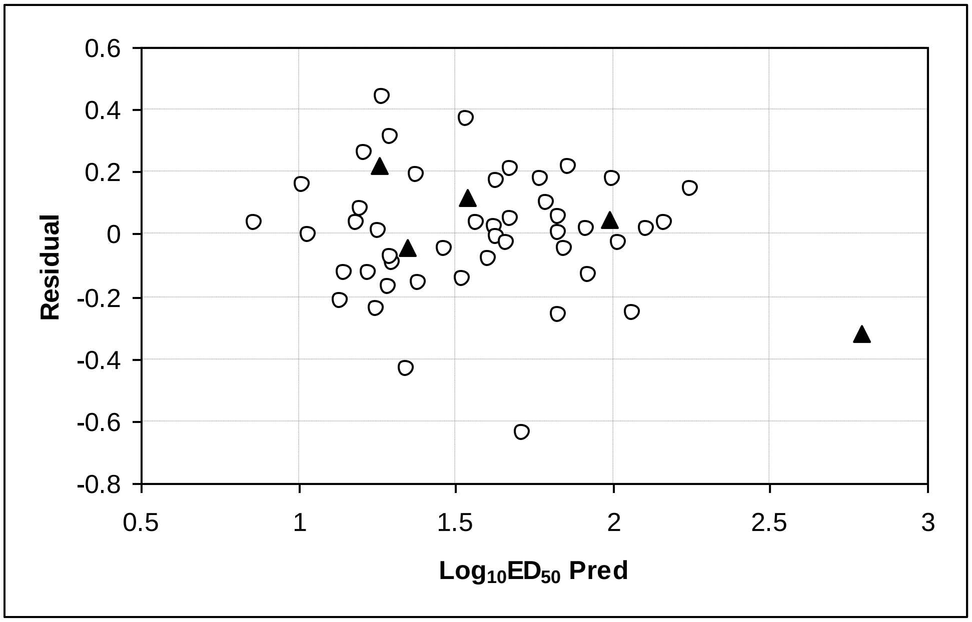

Figure 3S. Dispersion plot of the residuals for the ○ Calibration set and ▲ test set according to Eq. 5.

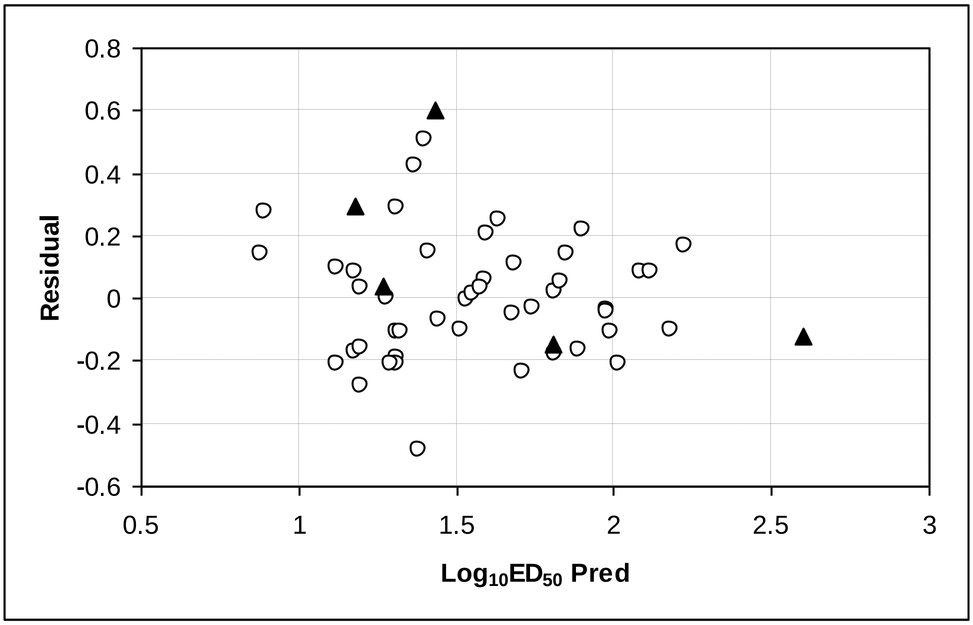

Supplement: Supplementary file 1 [file ijms-12-09354-s001.pdf]
